# Supplementary material for: Cardiac implantable electronic devices’ longevity: A novel modelling tool for estimation and comparison
Source: PLoS One. 2025 Sep 29;20(9):e0333195. doi: 10.1371/journal.pone.0333195 (PMC12478916; doi:10.1371/journal.pone.0333195)
Supplement: S2 File — (DOCX) [file pone.0333195.s002.docx]

**Supplementary Materials 2**

1. **Power consumption index rational**

The concept of Power consumption index (PCI) aims to address fundamental hurdles inherent to the concept of cardiac implant device longevity.

While energy consumption is well described by current (I), most physicians are not familiar with its concept and related values, or its impact on longevity which also depends on the reservoir of energy (the battery capacity). A device with C=1Ah and current consumption I=7,6 µA will have the same life span as another device with C=2 Ah and I=15,2 µA.

Moreover, longevity is not linear with current but it is proportional to battery capacity and an inverse to current (L=C/I). As a consequence of this non-linearity, the impact of each component of currents (background current, pacing current, etc…) on longevity is not proportional and therefore less intuitive.

The concept of Power consumption index PCI = t x I/C (where t is a constant equal to 1 hour) is designed to address these points:

1. The PCI takes into account the share of energy drained from the reservoir (C) by the current I over a specific timeframe (t).
2. Unlike current, this energy consumption index is an absolute concept without unit.
3. Finally, because PCI is linear with current it can be split according to each current contribution.

The table below (Longevity and PCI) gives indicative current value depending on battery capacity:

| Longevity (years) | 5y | 7,5y | 10y | 12,5y | 15y |
| --- | --- | --- | --- | --- | --- |
| PCI | 22,8 | 15,2 | 11,4 | 9,1 | 7,6 |
| I (µA) if C=0,8 Ah | 18,26 | 12,18 | 9,13 | 7,31 | 6,09 |
| **I (µA) if C=1Ah** | **22,8** | **15,2** | **11,4** | **9,1** | **7,6** |
| I (µA) if C=1,5 Ah | 34,25 | 22,83 | 17,12 | 13,70 | 11,42 |

The PCI is equivalent to the current if C=1Ah. A lower (higher) capacity with a lower (higher) current would achieve the same PCI.

Longevity is inversely related to PCI, (L=1/PCI if t=1hour)(see figure 1).

1. **Power consumption and nominal longevities**

Based on pacing current formulas and the current needed for each configuration, the PCI and the nominal longevity can be estimated for each device (see table below) under the following assumptions:

- For conventional pacemakers, the settings considered were: basic rate of 60bpm, pacing output at 2.5V, pulse duration: 0.4ms and impedance: 500 ohms for both A&V. For VVI pacemakers, ventricular pacing: 90% and for dual chamber pacemakers, atrial pacing: 70% for SND, 30% for AVB (51% on average) and ventricular pacing assumptions accounted for the difference between AAI/DDD mode and other RVP algorithms (29% vs 47%).

Options such as sensor, IEGM storage and remote (2 transmissions/year) are reported in Figure 2.

- For CRT-P, BIV was the standard pacing mode with alternative options such as aCRT^TM^ or MPP^TM^ pacing.
- For leadless VVI and DDD pacemakers, settings used in the model were: basic rate: 60bpm, pulse duration: 0.25ms for Micra™ and 0.4 ms for Aveir™, impedance: ~600 ohms for ventricle and ~300 ohms for the atrium. Pacing outputs were not reported in studies and two options were considered: 1.5V or 2.5V reflecting the level of confidence in lowering output (thresholds observed were typically low: 1.25V at implant and 0.75V weeks after). Pacing percentages were the same as for conventional pacemakers. Hysteresis mode was applied for DDD.

**PCI and nominal longevities**

**Evolution**

|  | | |  | | | Power consumption index split (PCI) | | | Longevity (years) | | |
| --- | --- | --- | --- | --- | --- | --- | --- | --- | --- | --- | --- |
|  | Generation | Background | Pacing | Sensor | Ext. IEGM | FUP | Remote | Total wo ext. IEGM | Total  w  ext. IEGM | wo ext. IEGM | w.  ext. IEGM |
| VVI | Previous | 10,4 | 2,4 | 0,8 | 1,7 | 0,1 | 0,0 | 13,7 | 15,4 | 8,8y | 8,3y |
| VVI | New | 6,9 | 1,9 | 0,8 | 0,2 | 0,1 | 0,9 | 10,6 | 10,8 | 11,0y | 10,8y |
| DDD | Previous | 9,4 | 1,8 | 0,7 | 2,1 | 0,1 | 0,2 | 12,2 | 14,3 | 9,9y | 9,0y |
| DDD | New | 7,6 | 1,7 | 0,7 | 0,2 | 0,1 | 0,8 | 11,0 | 11,2 | 10,7y | 10,5y |
| CRT-P | Previous | 7,9 | 3,8 | 0,7 | 1,4 | 0,1 | 0,0 | 12,5 | 13,9 | 9,2y | 8,3y |
| CRT-P | New | 7,8 | 4,3 | 0,8 | 1,0 | 0,1 | 1,1 | 14,1 | 15,0 | 8,3y | 7,8y |

**PCI and longevity model sensitivity analysis**

Whilst in early pacemaker generations, longevity calculations were presented in pacemaker user manuals with an estimate of margin of error, this has not been the case for almost a decade. Nevertheless, some residual variability may be related to technical features (battery capacity C, current components I_background_, I_pacing_,..). A sensitivity analysis can be performed to assess the impact on nominal longevity.

We have considered each of these in turn:

- **Battery**: Even if there is no fault, each battery varies compared with each other battery. Variations are anticipated by suppliers who set the Capacity at ERI accordingly. Battery voltage is followed up by generator interrogation during device life. For longevity calculation, the battery voltage is assumed to be constant (which is a proxy) and can only be checked for Bsc devices (where the data is available via the longevity website calculator). According to Bsc, variations are less than 1% (data is not disclosed by others).
- **I_background_** : the model shows that for current generation device there is -10% - 1% difference between modelled and declared current background. We assume a 5% variation for current background
- **I_pacing_ :** the consistency of pacing current is analyzed in Appendix 1 and 3 (Pacing current). We show that despite differences among models, the pacing current is generally consistent and robust despite different pacing configurations. The variations we found were limited to around 5.4 – 6%. In our model we therefore also assumed a 5% variation for the current of pacing.
- **I_IEGM_ , I_sensor,_ I_remote_ :** there are no available data on variability. Typically however, the variation is higher when nominal values are lower. Therefore we assume that variation is twice as much as **I_background_** or **I_pacing,_** which equates to around 10%

We have assumed that each one of these current component obeys an independent normal curve. Logically the total current also obeys a normal curve with its means being the sum of all means and its variances being the sum of all variances.

If we assume that capacity also fluctuates according to an independent normal curve the power consumption index (and its inverse longevity) can be modelled according to a ratio of two normal curves.

Subsequently deriving a sample size incorporates these assumptions: for a longevity span around 10 years, 5-10% variation accounts for a sigma around 0,5-1 year. For the calculation we assume sigma cannot exceed 1 year and sample size simulation accordingly (the sample size estimated is N = (1,96/0,01)^2 = 38416 close to 40000 samples).

The sensitivity analysis was programmed under Python with 40000 iterations for each device.

The table below reports the results of simulation:

Standard deviation is 3-4% across all devices. The 95% CI is tight as a consequence of the sample size (40,000).

When comparing device life span on a limited (clinically practical) sample size, the 95% confidence interval would be around 1,96 x [σ=0,20-0,50] / (2)^^0,5^ which is equivalent to 0,3-0,7 years. This explains why variations can be observed in the clinic setting despite similar settings for the same cardiac implant device. However, once the sample size reaches 100 for example, the 95% confidence interval is 1,96 x [σ=0,20-0,50] / (100)^^0,5^, equivalent to 0,04-0,10 years. Differences across devices can therefore be more noticeable in the routine clinical setting or in national registries where the sample size is smaller but when using modeling or a large national or international registry, the 95% CI is considerably tighter.

1. **Survival curves generated by the Monte-Carlo modelling**

**These are based upon a series of clinical assumptions:**

1. The devices settings used in the analysis are based on clinical data which are reported 2-3 years post implant.
2. Thresholds and outputs are typically stable 6 months post implant (thresholds typically vary very little from then apart from the relatively rare cases of lead dislodgment or lead fracture).
3. The majority of patients (>80%) do not need frequent reprogramming,^[[1]](#endnote-1)^ (which underlies the acceptance that remote follow-up is a reasonable way to provide pacemaker monitoring and that most alerts relate to atrial fibrillation rarely leading to programming intervention.
4. The main change after 2-3 years is the evolution of ventricular pacing percentage (the consequence of AV node disease progression). This again is based upon clinical observations:
   1. PR intervals rarely extend in patients with sinus node disease.^[[2]](#endnote-2)^
   2. In patients with significant AV node disease, with high grade AV block, the percentage of pacing is high, stable and is rarely reduced with reprogramming.^[[3]](#endnote-3)^
   3. In a population of people with intermittent AVB, the percentage of pacing increases at a predictable 1,5% increase per year.^[[4]](#endnote-4)^

As a result, the only changing variable in the model is the proportion of RV pacing and this is only for those patients with moderate AV blocks. In such cases, it is also clinically plausible that the percentage of pacing would depend on the use of RV pacing avoidance algorithms about which there are published data. Therefore, fortunately, inn this situation, we can use information from the literature in the model.

1. A small proportion of patients with a high percentage of pacing in whom there is a clinical or echocardiographic deterioration will undergo a change of system to CRT. This rarely occurs electively or opportunistically at generator end-of-life such that these patients are not considered in product survival curves (or Kaplan Meier).
2. For Medtronic CRT-P devices with Adaptive CRT, we have assumed that the percentage of RV pacing is that at 2-3 years (and not at implant).

Based upon these assumptions, we have assumed that, with the exception of those with intermittent AV block, settings at 2-3 years are stable and that the present approach is more clinically appropriate and likely to be more reliable than a Markov model since the probability of ‘transition’ would have been entirely heuristic given the lack of supporting literature. Such a (Markov) model would have added significant complexity, removed the clinically relevant points above and made the model much less accessible for clinicians.

**Settings and distribution used for Monte-Carlo simulations**

**Conventional single and dual pacemakers**

|  | **SND** | **Inter AVB** | **AVB perm** | **Distribution** |
| --- | --- | --- | --- | --- |
| V lead Z (Ω) | 500 ± 120 | 500 ± 120 | 500 ± 120 | Normal |
| V width (ms) | 0.35 [0.35; 0.35] | 0.35 [0.35; 0.35] | 0.35 [0.35; 0.35] | Constant |
| V output (V) | 2.5 [2.5; 3.5] | 2.5 [2.5; 3.5] | 2.5 [2.5; 3.5] | Normal |
| V output-LBBAP (V) | 2x 0,7 ± 0,24 | 2 x 0,7 ± 0,24 | 2x 0,7 ± 0,24 | Normal |
| VP VVI (%) | 90 [83; 100] | 90 [83; 100] | 90 [83; 100] | Normal |
| VP AAI/DDD* (%) | 2.2 [0.0; 29.9] | 53.5 [1.7; 95.9] ** | 89.2 [24.0; 99.3] | Per quartile |
| VP Other RVP* (%) | 2.2 [0.0; 29.9] | 97.9 [81.0; 99.6] | 98.2 [83.4; 99.6] | Per quartile |
| A lead Z *(Ω) | 450 ± 120 | 450 ± 120 | 450 ± 120 | Normal |
| A width *(ms) | 0.35 [0.35; 0.35] | 0.35 [0.35; 0.35] | 0.35 [0.35; 0.35] | Constant |
| A output *(V) | 2.5 [2.5; 3.5] | 2.5 [2.5; 3.5] | 2.5 [2.5; 3.5] | Normal |
| AP* (%) | 70.1 [30.5; 92.0] | 27.5 [9.0; 64.5] | 43.7 [2.6; 73.5] | Per quartile |

Legend : median [Q1; Q3], * specific to dual chamber pacemaker / pacing outputs are not reported yet for LBBAP pacing and are assumed to be twice as high as threshold value. ** Non fixed variable

**Single and dual chamber leadless pacemakers**

|  | **SND** | **Inter AVB** | **AVB perm** | **Distribution** |
| --- | --- | --- | --- | --- |
| V lead Z (Ω) | 627± 100 | 627± 100 | 627± 100 | Normal |
| V width (ms) | 0,24 ^M^ / 0,4 ^A^ | 0,24 ^M^ / 0,4 ^A^ | 0,24 ^M^ / 0,4 ^A^ | Constant |
| V output (V) | 2.5 [2.5; 3.5]  or 2x 0,75±0,5* | 2.5 [2.5; 3.5]  or 2x0,75 ±0,5* | 2.5 [2.5; 3.5]  or 2x0,75± 0,5* | Normal |
| VP VVI (%) | 90 [83; 100] | 90 [83; 100] | 90 [83; 100] | Normal |
| VP RVP* (%) | 2.2 [0.0; 29.9] | 97.9 [81.0; 99.6] | 98.2 [83.4; 99.6] | Per quartile |
| A lead Z (Ω)* | 315 ± 5 ^A^ | 315 ± 5 ^A^ | 315 ± 5 ^A^ | Normal |
| A width (ms)* | 0,4 ^A^ | 0,4 ^A^ | 0,4 ^A^ | Constant |
| A output (V)* | 2.5 [2.5; 3.5]  or 2x 0,75±0,5* | 2.5 [2.5; 3.5]  or 2x 0,75±0,5* | 2.5 [2.5; 3.5]  or 2x 0,75±0,5^*^ | Normal |
| AP (%)* | 70.1 [30.5; 92.0] | 27.5 [9.0; 64.5] | 43.7 [2.6; 73.5] | Per quartile |

Legend : median [Q1; Q3], * specific to dual chamber pacemaker (RVP is programmed with AV hysteresis mode), pacing conditions are selected according to clinical review for each leadless (^M^: Micra, ^A^ Aveir). See references : 17, 18, 19, 20. Comment: pacing thresholds are usually low 0.82 ± 0.8 (atrial) and 0.6 ± 0.5 (ventricle). Clinical studies do not report pacing outputs; hence two pacing output scenarios are considered.

**Cardiac resynchronisation therapy pacemakers**

|  |  | **Distribution** |
| --- | --- | --- |
| V lead Z (Ω) | 500 ± 120 | Normal |
| V width (ms) | 0.35 [0.35; 0.35] | Constant |
| V output (V) | 2.5 [2.5; 3.5] | Normal |
| VP BIV (%) | 95.1% ± 10.5% | Normal |
| VP Adapt CRT(%)* | 50.9% ± 15% | Normal |
| A lead Z (Ω) | 450 ± 120 | Normal |
| A width (ms) | 0.35 [0.35; 0.35] | Constant |
| A output (V) | 2.5 [2.5; 3.5] | Normal |
| AP (%) | 50% ± 15% | Per quartile |
| LV lead Z (Ω) | 745±325 | Normal |
| LV width (ms) | 0.35 [0.35; 0.35] | Constant |
| LV output (V) | 2.5 [1.7; 3.3] | Normal |
| LV BIV (%) | 95.1% ± 10.5% | Per quartile |

Same conditions as for conventional PM for A et V; LV conditions are extracted from literature (and specifically from Adaptive CRT trial for (*))

**Survival curves generated for previous generation devices**

| **VVI** | **DDD(R)** |
| --- | --- |
| 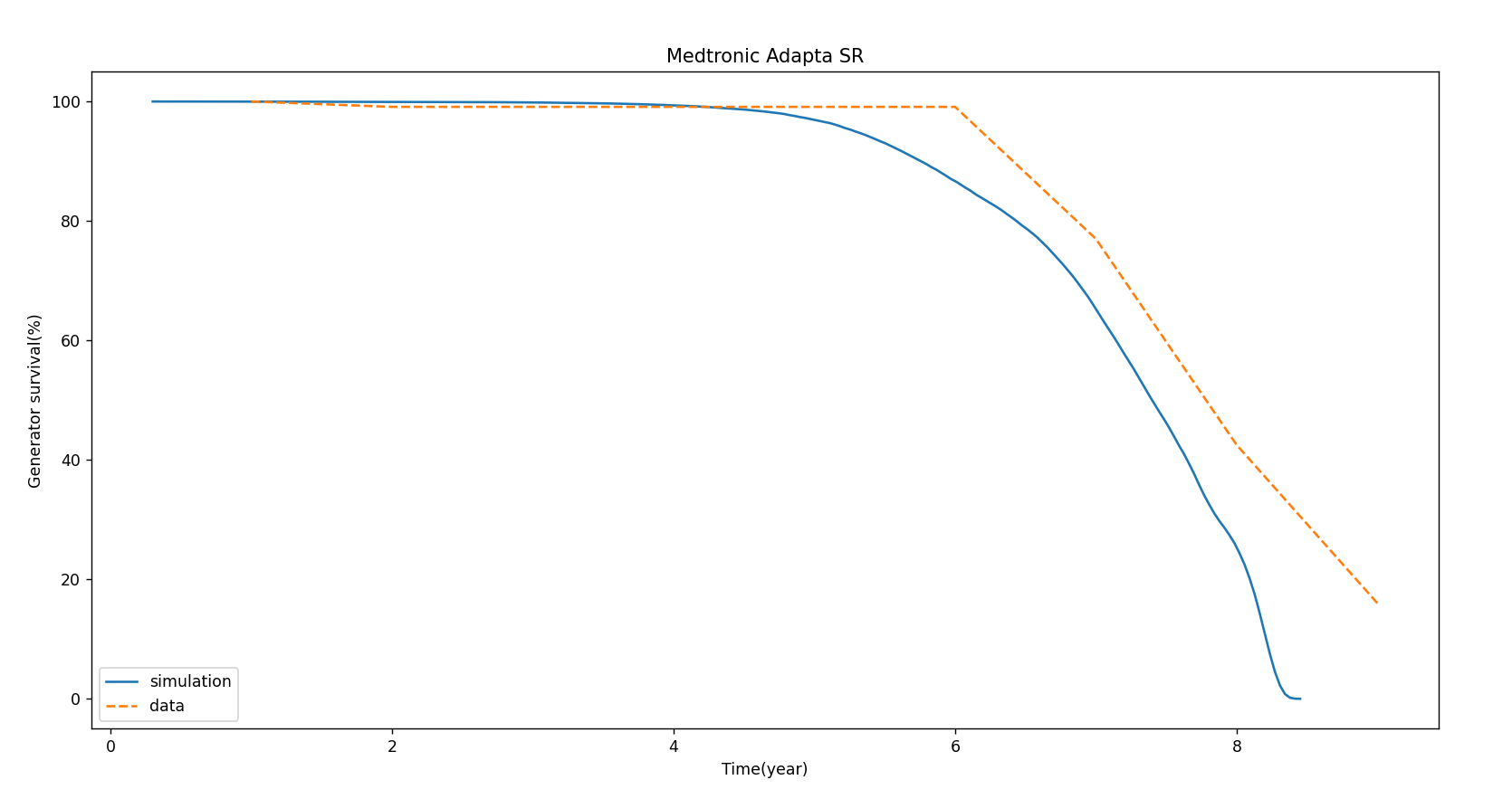 | 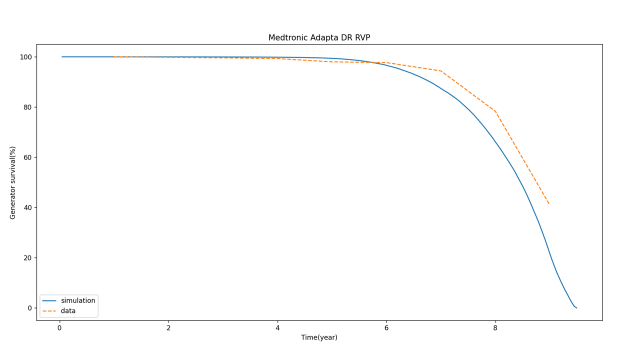 |
| 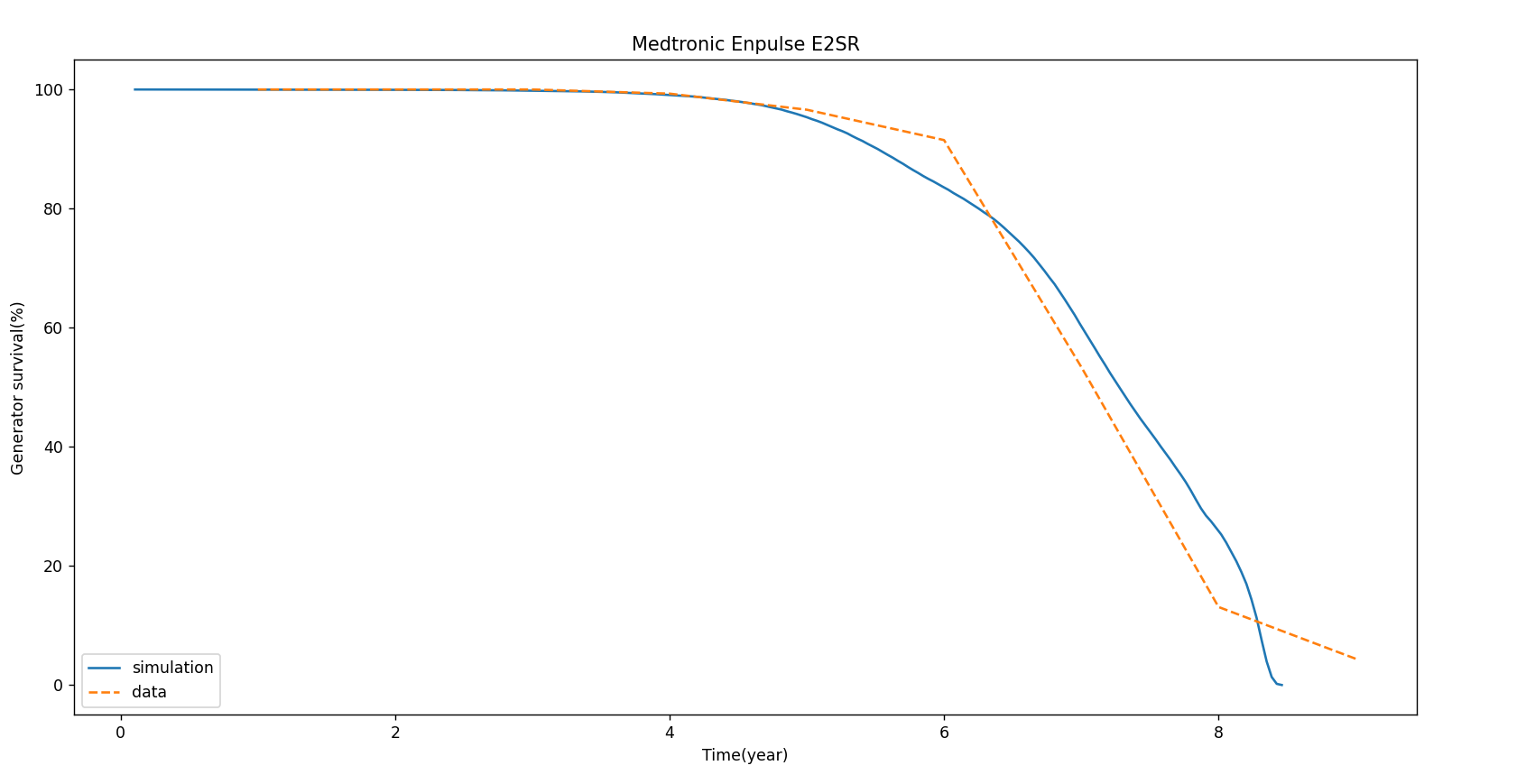 | 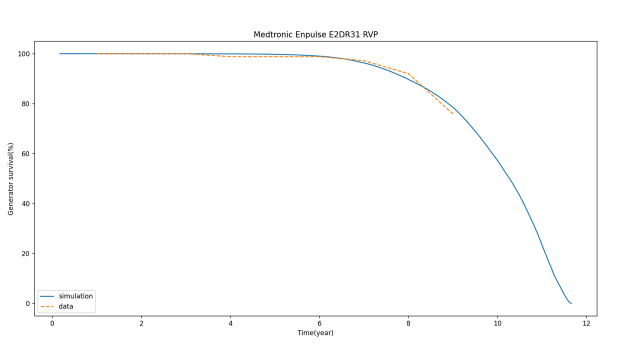 |
| 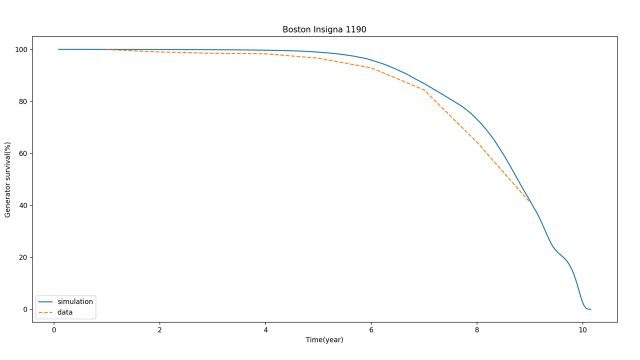 | 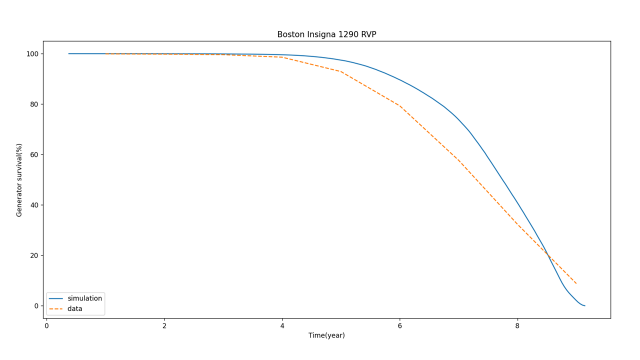 |
| 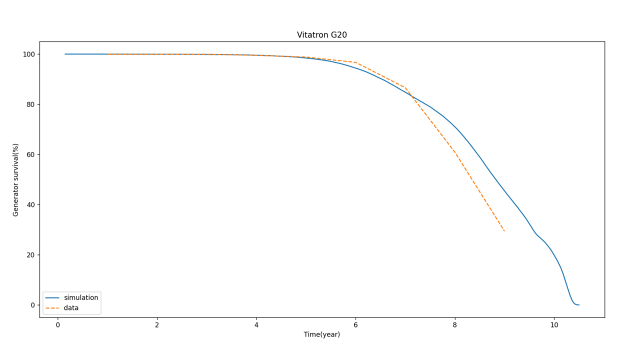 | 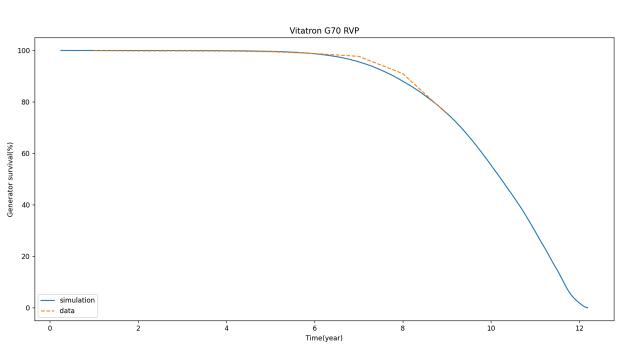 |
| 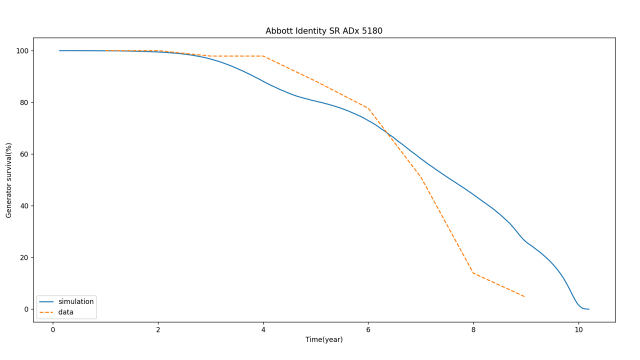 | 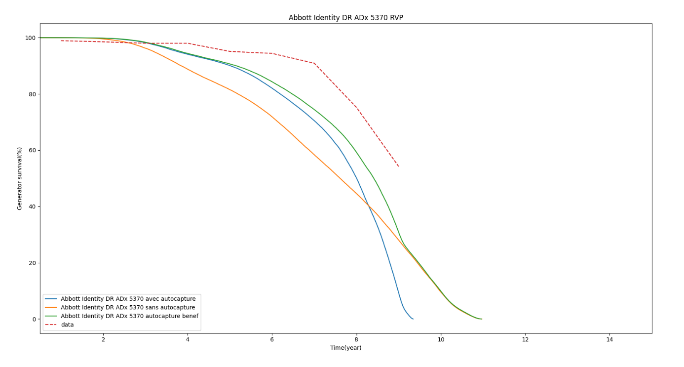 |

| **VVI** | **DDD(R)** |
| --- | --- |
| **NA** | 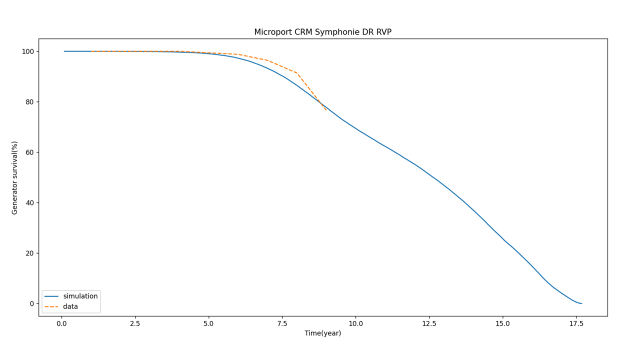 |

**Impact of settings for previous generation standard pacemaker devices**

| **Comments** | **DDD(R)** |
| --- | --- |
| With standard settings, the modeled survival curve for the Medtronic Enrythm DR with RV pacing avoidance activated, significantly deviates from all real life data. The only source of explanation is the use of IEGM storage (simulation assumes 1/3 pts without IEGM, 1/3 with IEGM, 1/3 with extended IEGM) | 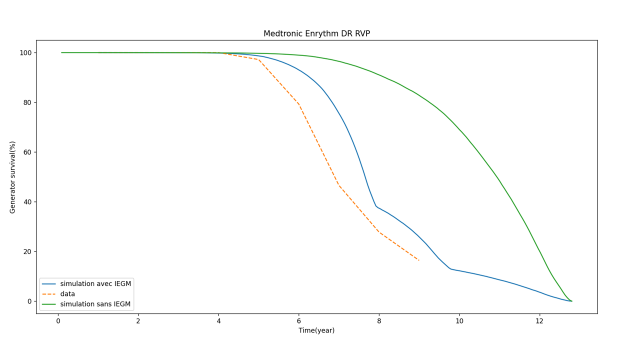 |
| With standard settings, the modeled survival curve of the Biotronik Evia DR-T deviates from real life data. Onn the other hand, the modelled survival curve with automatic threshold management on, matches real life data well. | 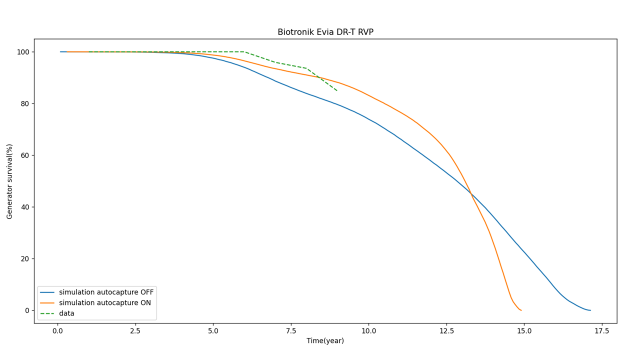 |
| With standard settings, the modeled survival curve of the Abbott Identity DR ADx deviates from real life data but with Auto capture^TM^ activated better matches the Swedish registry data.  The chart reports 2 scenarios: Auto capture™ activated for all patients versus Auto capture™ activated only if it improves longevity (i.e. the reduction of current drain via lower output was superior to the energy cost of Auto capture™) | 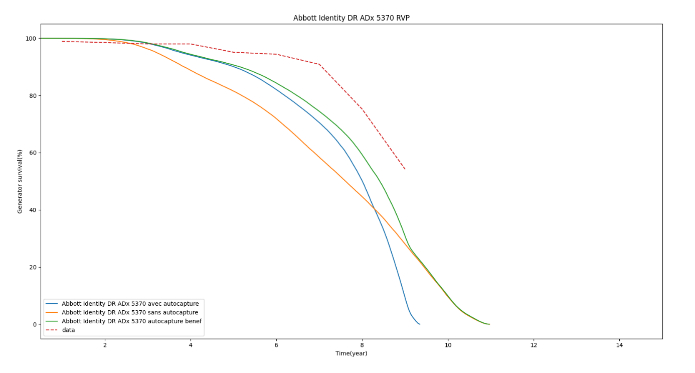 |

**Survival curves generated for CRT-P devices**

| **LV 2.5 +/-0.8V** 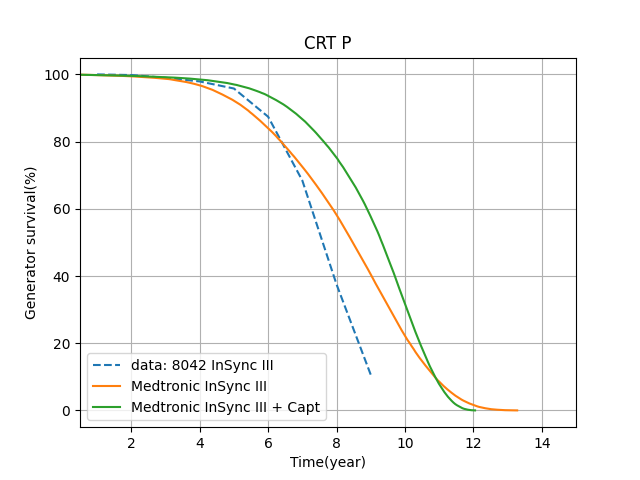 | **LV 3.5 +/-0.8V** 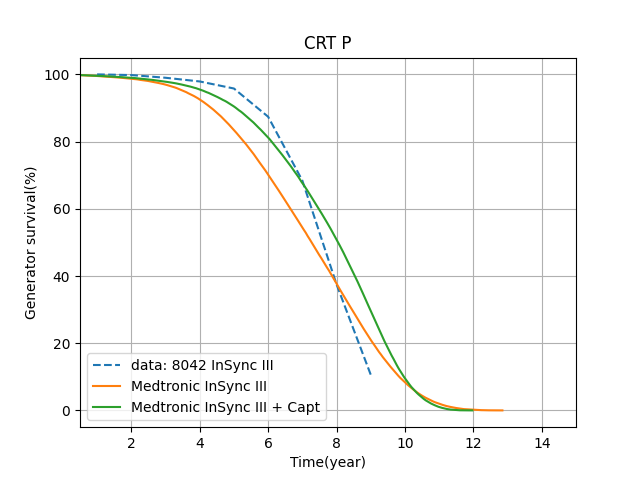 |
| --- | --- |

| 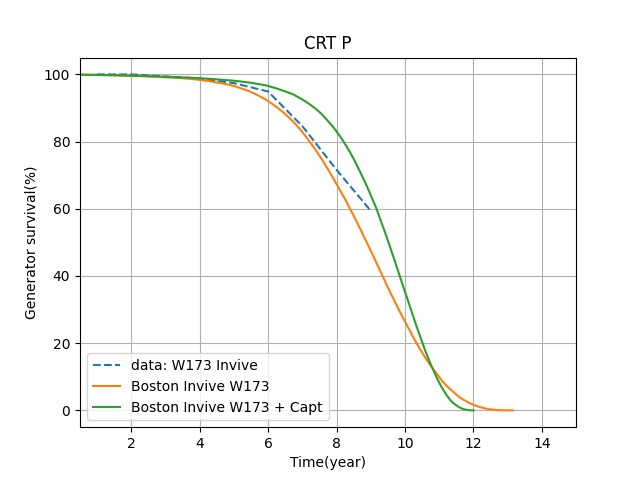 | 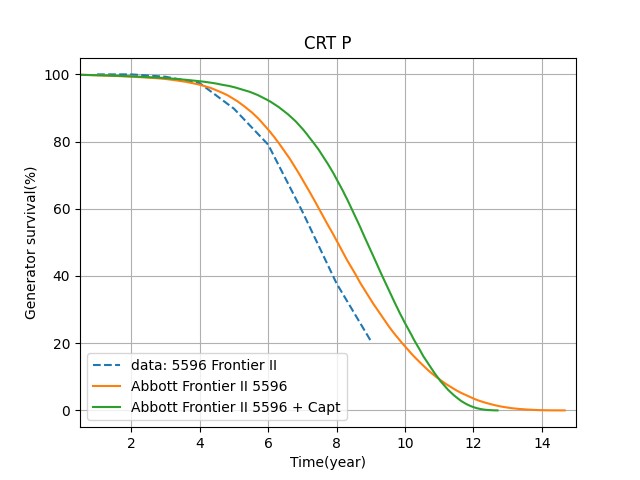 |
| --- | --- |
| 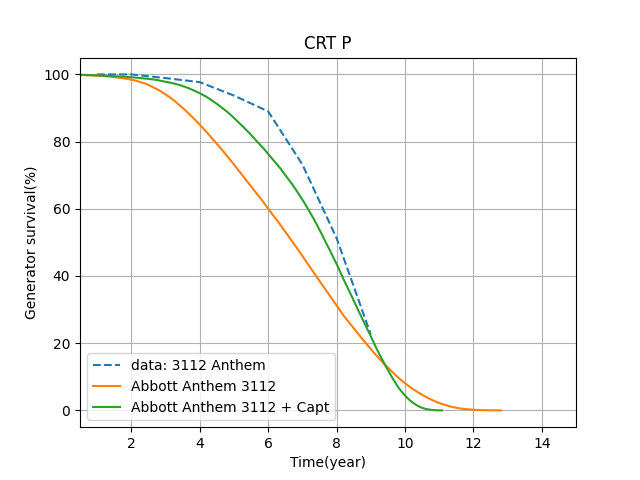 |  |

**Impact of settings for current generation devices**

**Impact of Automatic capture^TM^ on longevity**


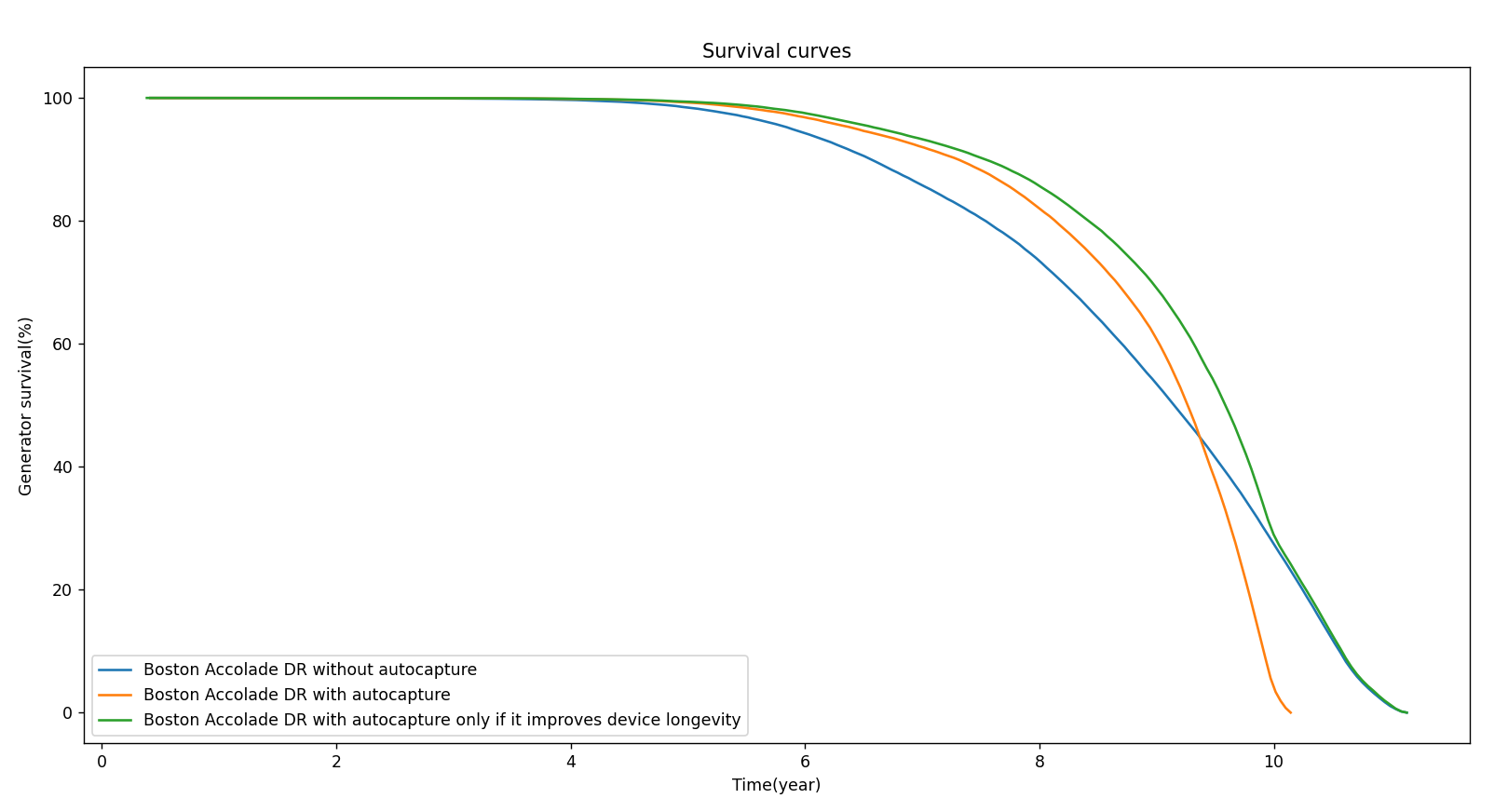


Assuming 1 μA energy cost for the algorithm and an ideal output of 1 V.

**Impact of RVP algorithm on longevity:**


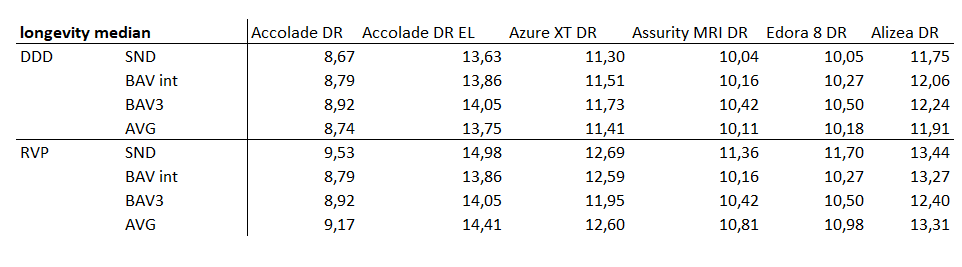


Median longevity is derived from the simulation of survival curve as described.

Note: in the modeling, RVP avoidance algorithms for intermittent AVB are only applied for those populations for which there is evidence of reduction of VP (MVP^TM^ ,Safe^TM^) and by extension to similar AAI/DDD mode (Btk).

**Impact of remote monitoring and downloads**

Standard settings with the activation of remote monitoring.


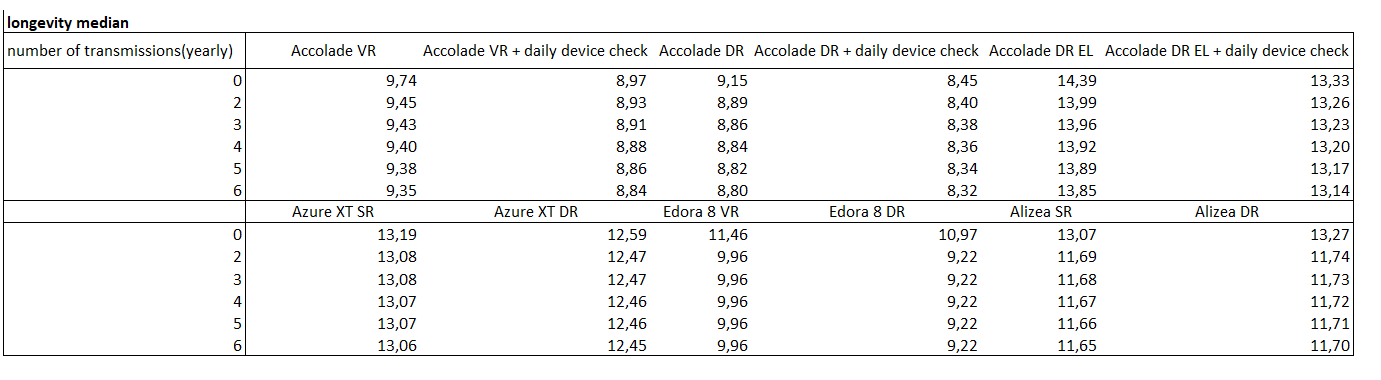


**References**

1. Udo EO, van Hemel NM, Zuithoff NP, Barrett MJ, Ruiter JH, Doevendans PA, Moons KG. Incidence and predictors of pacemaker reprogramming: potential consequences for remote follow-up. *Europace* 2013;**15:**978-83. doi: 10.1093/europace/eut002 [↑](#endnote-ref-1)
2. Thibault B, Ducharme A, Baranchuk A, Dubuc M, Dyrda K, Guerra PG, Macle L, Mondésert B, Rivard L, Roy D, Talajic M, Andrade J, Nitzsché R, Khairy P; CAN‐SAVE R Study Investigators. Very Low Ventricular Pacing Rates Can Be Achieved Safely in a Heterogeneous Pacemaker Population and Provide Clinical Benefits: The CANadian Multi-Centre Randomised Study-Spontaneous AtrioVEntricular Conduction pReservation (CAN-SAVE R) Trial. *J Am Heart Assoc* 2015;**4:**e001983. doi: 10.1161/JAHA.115.001983 [↑](#endnote-ref-2)
3. Stockburger M, Defaye P, Boveda S, Stancak B, Lazarus A, Sipötz J, Nardi S, Rolando M, Moreno J. Safety and efficiency of ventricular pacing prevention with an AAI-DDD changeover mode in patients with sinus node disease or atrioventricular block: impact on battery longevity-a sub-study of the ANSWER trial. *Europace* 2016;**18:**739-46. doi: 10.1093/europace/euv358 [↑](#endnote-ref-3)
4. Auricchio A, Ellenbogen KA. Reducing Ventricular Pacing Frequency in Patients With Atrioventricular Block: Is It Time to Change the Current Pacing Paradigm? *Circ Arrhythm Electrophysiol* 2016;**9:**e004404. doi: 10.1161/CIRCEP.116.004404 [↑](#endnote-ref-4)
