# Supplementary material for: Cardiac implantable electronic devices’ longevity: A novel modelling tool for estimation and comparison
Source: PLoS One. 2025 Sep 29;20(9):e0333195. doi: 10.1371/journal.pone.0333195 (PMC12478916; doi:10.1371/journal.pone.0333195)
Supplement: Attachment — Submitted filename: Responses to reviewers PONE-D-25-20064 R1.doc [file pone.0333195.s005.doc]

Reviewer 1: Thank you for the helpful and constructive comments, the incorporation of which have considerably improved the manuscript. We have been through the comments in detail and responded as follows:

Thank you for the opportunity to review this manuscript. The authors present a novel and unified framework for evaluating battery consumption across all CIEDs, introducing the concept of the Power Consumption Index (PCI). This concept is impressive and has the potential to provide valuable insights into the characteristics of individual CIEDs.

Response: Thank you

1) In the Introduction, the authors refer to their previous work, which involves the inverse of device longevity, and define PCI as t × l / C. However, this explanation feels somewhat abrupt. Without a careful reading of the supplemental materials, it is difficult to understand the logical basis for this definition.

Response: Thank you for this comment, which has stimulated us to describe our concept in more detail in the introduction. The initial concept was based around the fact that longevity is nonlinearly related to current complicating the creation of the index and it’s validation. The initial article by Lau *et al* described the inverse of longevity (which is not intuitive). Subsequently, we considered an alternative was to describe longevity as an harmonic mean compared with current, but a harmonic mean is not as intuitive as a standard mean making this also too complex for regular use. Since these details are very challenging for a clinical paper we have added additional details into Supplementary Material 2 in a section entitled ‘Power consumption index rationale’ which clarifies the rational for this concept where we have expanded the description of the rationale as follows:

1. **Power consumption index rational**

The concept of Power Consumption Index (PCI) aims to address fundamental hurdles inherent to the concept of cardiac implant device longevity.

While energy consumption is well described by current (I), most physicians are not familiar with its concept and related values, or its impact on longevity which also depends on the reservoir of energy (the battery capacity). A device with C=1Ah and current consumption I=7,6 µA will have the same life span as another device with C=2 Ah and I=15,2 µA.

Moreover, longevity is not linear with current but it is proportional to battery capacity and an inverse to current (L=C/I). As a consequence of this non-linearity, the impact of each component of currents (background current, pacing current, etc…) on longevity is not proportional and therefore less intuitive.

The concept of Power consumption index PCI = t x I/C (where t is a constant equal to 1 hour) is designed to address these points:

A) The PCI takes into account the share of energy drained from the reservoir (C) by the current I over a specific timeframe (t).
B) Unlike current, this energy consumption index is an absolute concept without unit.
C) Finally, because PCI is linear with current it can be split according to each current contribution.

The table below (Longevity and PCI) gives indicative current value depending on battery capacity:

| Longevity (years) | 5y | 7,5y | 10y | 12,5y | 15y |
| --- | --- | --- | --- | --- | --- |
| PCI | 22,8 | 15,2 | 11,4 | 9,1 | 7,6 |
| I (µA) if C=0,8 Ah | 18,26 | 12,18 | 9,13 | 7,31 | 6,09 |
| **I (µA) if C=1Ah** | **22,8** | **15,2** | **11,4** | **9,1** | **7,6** |
| I (µA) if C=1,5 Ah | 34,25 | 22,83 | 17,12 | 13,70 | 11,42 |

The PCI is equivalent to the current if C=1Ah. A lower (higher) capacity with a lower (higher) current would achieve the same PCI.

Longevity is inversely related to PCI, (L=1/PCI if t=1hour)(see figure 1).

2) Figure 2 is particularly impressive, as it demonstrates the variability in PCI for background current and other functions across different pacemaker models. Could the authors comment on the factors that contribute to differences in PCI for background current (I_background) between models, even among those in the same category and generation?

Response: Thank you, we agree that the data do look impressive. Regarding their excellent question about the factors that lead to different background current the Expert Reviewer is directed to the section in the results section on ‘Estimation of nominal longevities, using the PCI model’ (page 13) where we say:

‘Among the contemporary devices, Accolade™ SR/DR had the highest PCI, and thus the lowest estimated longevity (PCI of 13.5 and longevity of 8.5 years for SR, PCI of 14.2 and longevity of 8 years for DR). This is because this device had the highest I_background_ (10µA) as compared with other devices in the same category. On the other hand, Accolade™ DR EL, even with activated remote monitoring, had the lowest PCI (8.9), and thus the longest longevity (12.8 years), thanks to the high battery capacity at 1.6Ah.’

And

‘For the other devices, differences were primarily due to optional settings such as extended IEGM, sensor or remote monitoring.’

This section also includes information as follows:

‘Unlike conventional pacemakers, PCI related to pacing in leadless pacemakers accounted for more of the total PCI (40% on average, higher with 2.5V outputs). This is the consequence of a lower battery capacity and thus, a greater proportion of total available energy is required for pacing. Consequently, PCI and longevity significantly changed depending on pacing output assumptions (Aveir™ VVI: 15.1 years if at 1.5V *vs*. 10.6 years if at 2.5V; Micra™ VVI:10.2 years *vs*. 6.6 years, respectively; Aveir™ ventricular capsule DDD mode: 12.5 years *vs*. 10.6 years; Aveir™ atrial capsule DDD mode 7.3 *vs*. 5.4 years, respectively).’

We hope that this detail is sufficient for a clinical reader.

3) What are the potential clinical implications of the PCI concept for practitioners, beyond the conventional longevity estimates provided by manufacturers during device checks? How should clinicians apply the PCI framework when selecting pacemaker models within the same category?

Response: Using the PCI related to background current, physicians could ensure that they choose the devices with the best longevity for the features that they require and they can also make a decision on which items to deactivate. The lowest PCI related to background current means that more energy is ‘in the tank’ available for therapy. We have incorporated a reference to table 4, which now is updated to include practical recommendations. The table includes a variety of options that clinicians can use to try and extend battery life or personalise device prescription. In the Discussion section entitled ‘Battery capacity and background current’ we have directed the Reader to table 4 saying:

‘This is the purpose of the index proposed here. The PCI combines background current (i.e C/(365*24*10^^-6^ I_b_) and battery capacity and thereby the energy capacity (in years) available for pacing and programming options. Applying this criterion could contribute to personalized device selection, with a focus on device longevity for a wide range of patients (see table 4).’

Using these data, we have also proposed options for programming the output of leadless pacemakers to maintain battery longevity which are included in table 4.

Reviewer #2: Thank you for the helpful and constructive comments, the incorporation of which have considerably improved the manuscript. We have been through the comments in detail and responded as follows:

The study by Defaye et al. offers a novel and practical approach to estimating and comparing the longevity of CIEDs using a Power Consumption Index (PCI) derived from battery capacity and modeled current drain. The inclusion of diverse device types and manufacturers, combined with a large-scale Monte Carlo simulation, enhances the generalizability of the findings. Additionally, the validation of model outputs using data from the Swedish device registry further supports the study’s clinical relevance. However, there are several limitations related to the PCI model and the interpretation of its outputs that should be discussed further to improve the robustness and applicability of the findings.

Response: Thank you.

1. A major limitation of the model is that the authors assumed that device settings and current drain remain stable beyond 2–3 years. However, in practice, pacing thresholds, lead performance, and device efficiency can change over time, and battery internal resistance increases as the device ages. These changes are especially relevant in long-lived or high-burden pacing scenarios and can significantly alter energy consumption over the device’s lifespan. The authors need to address how such non-linear and time-dependent variations might affect the validity of their model. Given these limitations, the PCI model may be more appropriate as a comparative framework for evaluating relative differences between devices, rather than as a precise predictor of absolute longevity.

Response: This is an important point and we are grateful to the reviewer for raising it. We have expanded the information in Supplementary Material 2 to add further explanation on this.

More specifically regarding the Reviewer’s insightful comment, we have clarified in the second section of Supplementary Material 2 why we only adjust for the proportion of RV pacing in a modest population of patients: namely those exclusively with intermittent AV block in whom RV pacing avoidance algorithms have the capacity to alter the amount of pacing, and for whom such data are available in the literature and in whom the capacity for progressive AV block is of relevance. This is not the case for patients with sinus node disease (in whom progressive AV block is rare) or for people with complete heart block where the proportion of RV pacing is unavoidable. We have added to the previous description of this into limitations in the manuscript as follows:

‘The literature review performed to retrieve standard pacemaker parameters provided information on the average settings of devices. As RVP avoidance algorithms were not consistently investigated through clinical trials, percentages of pacing achieved were assumed to be identical according to type (AAI/DDD mode, hysteresis mode such as SAV+^®^, VIP^®^) for SND patients. On the other hand, the impact of AAI/DDD modes (MVP^®^, SafeR^®^, Vp suppression^®^) for intermittent AVB patients was investigated in clinical trials and therefore could be considered in the model. For these patients we considered a 1.5% increase per year of ventricular pacing to factor progressive A-V node disease over time. Apart from this group, we had to assume that settings are stable beyond 2-3 years as we lacked clinical data over a longer time frame. For this assumption, we could have chosen another approach and built a Markov model to factor changes across time, but the probability of transition would have been ‘heuristic’ since such data are not available in literature. This would have added significant complexity to our model without changing our overall point and making the present manuscript less accessible.’

To provide further clarification we have also included greater commentary into Supplementary Materials 2 regarding this point where was also comment on the rate of progression (or not) and outline why we did not use a Markov model where we now say:

2. ‘**Survival curves generated by the Monte-Carlo modelling**

**These are based upon a series of clinical assumptions:**

A) The devices settings used in the analysis are based on clinical data which are reported 2-3 years post implant.
B) Thresholds and outputs are typically stable 6 months post implant (thresholds typically vary very little from then apart from the relatively rare cases of lead dislodgment or lead fracture).
C) The majority of patients (>80%) do not need frequent reprogramming,^[1]^ (which underlies the acceptance that remote follow-up is a reasonable way to provide pacemaker monitoring and that most alerts relate to atrial fibrillation rarely leading to programming intervention.
D) The main change after 2-3 years is the evolution of ventricular pacing percentage (the consequence of AV node disease progression). This again is based upon clinical observations:
   a. PR intervals rarely extend in patients with sinus node disease.^[2]^
   b. In patients with significant AV node disease, with high grade AV block, the percentage of pacing is high, stable and is rarely reduced with reprogramming.^[3]^
   c. In a population of people with intermittent AVB, the percentage of pacing increases at a predictable 1,5% increase per year.^[4]^

As a result, the only changing variable in the model is the proportion of RV pacing and this is only for those patients with moderate AV blocks. In such cases, it is also clinically plausible that the percentage of pacing would depend on the use of RV pacing avoidance algorithms about which there are published data. Therefore, fortunately, inn this situation, we can use information from the literature in the model.

E) A small proportion of patients with a high percentage of pacing in whom there is a clinical or echocardiographic deterioration will undergo a change of system to CRT. This rarely occurs electively or opportunistically at generator end-of-life such that these patients are not considered in product survival curves (or Kaplan Meier).
F) For Medtronic CRT-P devices with Adaptive CRT, we have assumed that the percentage of RV pacing is that at 2-3 years (and not at implant).

Based upon these assumptions, we have assumed that, with the exception of those with intermittent AV block, settings at 2-3 years are stable and that the present approach is more clinically appropriate and likely to be more reliable than a Markov model since the probability of ‘transition’ would have been entirely heuristic given the lack of supporting literature. Such a (Markov) model would have added significant complexity, removed the clinically relevant points above and made the model much less accessible for clinicians.’

It is important to recognize that the internal resistance of the battery increases as the device ages. This is relevant for the calculation of residual longevity months or years after implant and is used by the manufacturer to estimate residual longevity during follow-up. Whilst total estimated longevity is based upon the battery capacity at ERI, manufacturers consider the evolution of battery discharge and increase of resistance in combination (based on simulations provided by the battery manufacturer) to carefully select the point of ‘ERI’.

2. The authors estimated background and pacing currents by using regressing based on manufacturer-reported device longevities. However, these reported longevities are often rounded, modeled, or based on internal assumptions that are not publicly transparent. As a result, even small uncertainties in input longevity, such as a difference of ±0.5 years, can lead to noticeable changes in the inferred current drain and the resulting PCI values. I suggest the authors perform a sensitivity analysis to examine how small variations in input longevity values, for example within a range of ±0.5 to ±1 year can impact the inferred current drain and the resulting PCI. This further sensitivity analysis would help quantify the robustness of the model, clarify which device categories or settings are more sensitive to input variability, and improve the overall transparency and reliability of the model.

Response: We are grateful for the Expert Reviewer’s comments and we agree entirely that even a small variation of longevity could be clinically important. However we would like to comment in more detail on the proposal that we carry out a sensitivity analysis.

Firstly, although there is electrochemically the possibility of variation, the PCI calculation and the longevities in the user manuals present longevity calculations in the setting of fixed parameters (frequency, impedance, output, pacing). Hence, the critical piece of the puzzle that contributes the greatest variability for any pacemaker is actually the patient and we have already included this factor into the Monte-Carlo simulation (see above and Supplementary Materials 2).

Further variability in nominal longevity calculation can therefore, as suggested by the Expert Reviewer, only be related to the technical features (battery capacity C, and the current components I_background_, I_pacing_ and the specific algorithms such as RVP which modify I_pacing_). We have considered each of these in turn in Supplementary Materials 2:

**PCI and longevity model sensitivity analysis**

Whilst in early pacemaker generations, longevity calculations were presented in pacemaker user manuals with an estimate of margin of error, this has not been the case for almost a decade. Nevertheless, some residual variability may be related to technical features (battery capacity C, current components I_background_, I_pacing_,..). A sensitivity analysis can be performed to assess the impact on nominal longevity.

We have considered each of these in turn:

- **Battery**: Even if there is no fault, each battery varies compared with each other battery. Variations are anticipated by suppliers who set the Capacity at ERI accordingly. Battery voltage is followed up by generator interrogation during device life. For longevity calculation, the battery voltage is assumed to be constant (which is a proxy) and can only be checked for Bsc devices (where the data is available via the longevity website calculator). According to Bsc, variations are less than 1% (data is not disclosed by others).
- **I_background_** : the model shows that for current generation device there is -10% - 1% difference between modelled and declared current background. We assume a 5% variation for current background
- **I_pacing_ :** the consistency of pacing current is analyzed in Appendix 1 and 3 (Pacing current). We show that despite differences among models, the pacing current is generally consistent and robust despite different pacing configurations. The variations we found were limited to around 5.4 – 6%. In our model we therefore also assumed a 5% variation for the current of pacing.
- **I_IEGM_ , I_sensor,_ I_remote_ :** there are no available data on variability. Typically however, the variation is higher when nominal values are lower. Therefore we assume that variation is twice as much as **I_background_** or **I_pacing,_** which equates to around 10%

We have assumed that each one of these current component obeys an independent normal curve. Logically the total current also obeys a normal curve with its means being the sum of all means and its variances being the sum of all variances.

If we assume that capacity also fluctuates according to an independent normal curve the power consumption index (and its inverse longevity) can be modelled according to a ratio of two normal curves.

Subsequently deriving a sample size incorporates these assumptions: for a longevity span around 10 years, 5-10% variation accounts for a sigma around 0,5-1 year. For the calculation we assume sigma cannot exceed 1 year and sample size simulation accordingly (the sample size estimated is N = (1,96/0,01)^2 = 38416 close to 40000 samples).

The sensitivity analysis was programmed under Python with 40000 iterations for each device.

The table below reports the results of simulation

Standard deviation is 3-4% across all devices. The 95% CI is tight as a consequence of the sample size (40,000).

When comparing device life span on a limited (clinically practical) sample size, the 95% confidence interval would be around 1,96 x [=0,20-P,50] / (2)^^0,5^ which is equivalent to 0,3-0,7 years. This explains why variations can be observed in the clinic setting despite similar settings for the same cardiac implant device. However, once the sample size reaches 100 for example, the 95% confidence interval is 1,96 x [=0,20-P,50] / (100)^^0,5^, equivalent to 0,04-0,10 years. Differences across devices can therefore be more noticeable in the routine clinical setting or in national registries where the sample size is smaller but when using modeling or a large national or international registry, the 95% CI is considerably tighter.

3. In the Monte Carlo simulation, the authors modeled 100,000 virtual patients with varying physiological and device-related parameters, including pacing burden, output voltage, lead impedance, algorithm usage, and settings such as IEGM and remote monitoring. However, the results are presented only as point estimates, without reporting summary statistics such as standard deviations, interquartile ranges, or 95% CI. I recommend the authors include these metrics to reflect the level of uncertainty in the estimates. This would clarify whether the differences in predicted longevity across devices are statistically meaningful or potentially fall within the range of expected variability. Without this information, comparative interpretations may be misleading.

Response: The sample size (100,000 virtual patients) was selected to match the typical sample size of product performance reports (PPRS) which aggregate worldwide data. Most publications related to longevity are based on much smaller sample size. This also true for Swedish Cardiac Implant Registry.^[5]^ The rational for such a sample size was to have the lowest 95% CI in order to have the best comparison between devices. The 95% CI survival curves are reported in a new supplementary data (supplementary material 3 Excel file). The 95% confidence intervals are extremely low, due to the size (100,000) of the simulated population, allowing a comparison across devices.

.

Reviewer #3: Thank you for the helpful and constructive comments, the incorporation of which have considerably improved the manuscript. We have been through the comments in detail and responded as follows:

1. The concept of PCI presents a clear advancement in the comparative assessment of CIED longevity. By attempting to unify device longevity estimation into a harmonized, simulation-validated model, this work contributes significantly to a longstanding gap in device transparency and comparability.

Response: Thank you.

Suggestion for enhancement: The manuscript would benefit from a deeper contextualization of PCI relative to past methods (e.g., specific critiques of vendor calculators and earlier power consumption models). This would clarify the incremental benefit of PCI beyond ‘simplification.’

Response: Thank you for this comment. We have expanded the supplementary information as follows:

3. **Power consumption index rational**

The concept of Power consumption index (PCI) aims to address fundamental hurdles inherent to the concept of cardiac implant device longevity.

While energy consumption is well described by current (I), most physicians are not familiar with its concept and related values, or its impact on longevity which also depends on the reservoir of energy (the battery capacity). A device with C=1Ah and current consumption I=7,6 µA will have the same life span as another device with C=2 Ah and I=15,2 µA.

Moreover, longevity is not linear with current but it is proportional to battery capacity and an inverse to current (L=C/I). As a consequence of this non-linearity, the impact of each component of currents (background current, pacing current, etc…) on longevity is not proportional and therefore less intuitive.

The concept of Power consumption index PCI = t x I/C (where t is a constant equal to 1 hour) is designed to address these points:

D) The PCI takes into account the share of energy drained from the reservoir (C) by the current I over a specific timeframe (t).
E) Unlike current, this energy consumption index is an absolute concept without unit.
F) Finally, because PCI is linear with current it can be split according to each current contribution.

The table below (Longevity and PCI) gives indicative current value depending on battery capacity:

| Longevity (years) | 5y | 7,5y | 10y | 12,5y | 15y |
| --- | --- | --- | --- | --- | --- |
| PCI | 22,8 | 15,2 | 11,4 | 9,1 | 7,6 |
| I (µA) if C=0,8 Ah | 18,26 | 12,18 | 9,13 | 7,31 | 6,09 |
| **I (µA) if C=1Ah** | **22,8** | **15,2** | **11,4** | **9,1** | **7,6** |
| I (µA) if C=1,5 Ah | 34,25 | 22,83 | 17,12 | 13,70 | 11,42 |

The PCI is equivalent to the current if C=1Ah. A lower (higher) capacity with a lower (higher) current would achieve the same PCI.

Longevity is inversely related to PCI, (L=1/PCI if t=1hour)(see figure 1).

2. The use of Monte-Carlo simulation based on a robust sample (100,000 virtual patients) and comparison with real-world registry data lends credibility to the PCI framework. However, the validation is partially limited due to assumptions required for missing data and registry limitations.

Suggestion: Include a sensitivity analysis of model assumptions (e.g., effects of varying remote monitoring or pacing algorithm adoption rates). This would strengthen confidence in generalizability.

Response: We acknowledge these limitations and it is true that a sensitivity analysis could address this point. There are 3 categories of parameters whose fluctuations can impact real life longevity: 1) technical factors (capacity or current); 2) patient population (clinical indication); 3) device programming. When it comes to technical factors, it is true that a sensitivity analysis (impact of fluctuation of technical factors on nominal longevity) was missing. This sensitivity analysis is now included in supplementary material 2. Standard deviation is close to 3-4% and 95%CI can be 0.04-0.10 years as soon as sample size reaches 100 (this could explain why suppliers do not report margin of error for nominal longevity in user manuals). When it comes to point 2 and point 3 there are interrelated (clinical reports disclose average programming of device per indication).

The large sample size of Monte-Carlo patient simulation (100,000) gives an extremely low CI (now included in supplementary material 3) and addresses the need to consider patient variability within our longevity model.

We now say in Supplementary Materials 2:

**PCI and longevity model sensitivity analysis**

Whilst in early pacemaker generations, longevity calculations were presented in pacemaker user manuals with an estimate of margin of error, this has not been the case for almost a decade. Nevertheless, some residual variability may be related to technical features (battery capacity C, current components I_background_, I_pacing_,..). A sensitivity analysis can be performed to assess the impact on nominal longevity.

We have considered each of these in turn:

- **Battery**: Even if there is no fault, each battery varies compared with each other battery. Variations are anticipated by suppliers who set the Capacity at ERI accordingly. Battery voltage is followed up by generator interrogation during device life. For longevity calculation, the battery voltage is assumed to be constant (which is a proxy) and can only be checked for Bsc devices (where the data is available via the longevity website calculator). According to Bsc, variations are less than 1% (data is not disclosed by others).
- **I_background_** : the model shows that for current generation device there is -10% - 1% difference between modelled and declared current background. We assume a 5% variation for current background
- **I_pacing_ :** the consistency of pacing current is analyzed in Appendix 1 and 3 (Pacing current). We show that despite differences among models, the pacing current is generally consistent and robust despite different pacing configurations. The variations we found were limited to around 5.4 – 6%. In our model we therefore also assumed a 5% variation for the current of pacing.
- **I_IEGM_ , I_sensor,_ I_remote_ :** there are no available data on variability. Typically however, the variation is higher when nominal values are lower. Therefore we assume that variation is twice as much as **I_background_** or **I_pacing,_** which equates to around 10%

We have assumed that each one of these current component obeys an independent normal curve. Logically the total current also obeys a normal curve with its means being the sum of all means and its variances being the sum of all variances.

If we assume that capacity also fluctuates according to an independent normal curve the power consumption index (and its inverse longevity) can be modelled according to a ratio of two normal curves.

Subsequently deriving a sample size incorporates these assumptions: for a longevity span around 10 years, 5-10% variation accounts for a sigma around 0,5-1 year. For the calculation we assume sigma cannot exceed 1 year and sample size simulation accordingly (the sample size estimated is N = (1,96/0,01)^2 = 38416 close to 40000 samples).

The sensitivity analysis was programmed under Python with 40000 iterations for each device.

The table below reports the results of simulation

Standard deviation is 3-4% across all devices. The 95% CI is tight as a consequence of the sample size (40,000).

When comparing device life span on a limited (clinically practical) sample size, the 95% confidence interval would be around 1,96 x [σ=0,20-P,50] / (2)^^0,5^ which is equivalent to 0,3-0,7 years. This explains why variations can be observed in the clinic setting despite similar settings for the same cardiac implant device. However, once the sample size reaches 100 for example, the 95% confidence interval is 1,96 x [σ=0,20-P,50] / (100)^^0,5^, equivalent to 0,04-0,10 years. Differences across devices can therefore be more noticeable in the routine clinical setting or in national registries where the sample size is smaller but when using modeling or a large national or international registry, the 95% CI is considerably tighter.

When it comes to the most impacting programming options (extended IEGM, beat to beat threshold algorithms and remote monitoring) they are investigated in terms of current (see supplementary materials 1) and product survival curve (see impact of settings on supplementary materials 2). Their impact on longevity can be dramatic on longevity (due to the energy cost of the algorithm and not because of changing patient factors).

3. The coverage across device types and manufacturers is commendable. The device-level analysis is precise and allows clinically relevant interpretations.
Suggestion: Consider organizing data in visual heatmaps (e.g., PCI vs. longevity by manufacturer and device type) to enhance accessibility.

Response: We are always grateful for suggestions on how to make the data more accessible but we are not sure that this suggestion will simplify the article and the message. In this case, given the number of figures and the amount of information already included, we would be grateful for the advice of the editorial team on whether a heat map would help.

4. The manuscript occasionally drifts into technical detail at the expense of clinical utility. While technically sound, the discussion should better highlight how clinicians could practically use PCI in device selection and programming.
Suggestion: Develop a decision-support table or tool prototype for clinicians, ideally as a supplementary file, demonstrating how PCI could inform real-world decisions.

Response: Thank you for this. Our priority has always been to balance technical accuracy with accessibility for clinicians and payers. Based on the reviewer’s comment, we have revised table 4 to provide practical recommendations. Moreover, the manuscript has been revised thoroughly for readability and clarity. Not all of the minor changes have been highlighted to avoid the whole text being highlighted.

| **Steps** | **Overall recommendations** | **Specific points** |
| --- | --- | --- |
| 1 | - For device selection, investigate PCI index related to current background (this insures that the device offers the best energy resource for therapy delivery once current background is factored) | - For conventional SR, DR, avoid Accolade™ SR/DR (non EL-version) and for CRT-P, prefer Visionist™, Percepta™ (+aCRT™) - For leadless auricular, anticipate early replacement for SND patients and then prefer leadless DDD for intermittent AVB |
| 2 | - Then, depending on patient profile, select device with adequate pacing options: - When DDDR is recommended, give priority to reduction of ventricular pacing - When VVI or CRT-P is required or when percentage of pacing with DR exceeds 60%, check capture algorithm is available to reduce pacing output (in particular for leadless) | - Use available RVPa for SND pts ; prefer AAI/DDD for both SNDs & inter. AVB - RV capture is inefficient if combined with aCRT™ |
| 3 | - Check need of additional options (IEGM additional storage, sensors..) | - Among options additional IEGM storage impacts the most longevity |
| 4 | - Carefully program remote follow-up and alert to insure relevant transmissions | - Anticipate “one off” energy cost for some devices (Edora™, Alizea™) - Avoid using inductive telemetry for leadless pacemaker |

5. The manuscript discloses relevant conflicts of interest transparently. However, due to multiple affiliations with device manufacturers, independent validation or collaboration with a neutral third party would improve perceived impartiality.

Response: Thank you for this comment. We would like to assure the Expert Reviewer that, as can be seen by the entirely technical and unbiased analysis and the discussion of devices’ longevity without prejudice by manufacturer or device, there have been no external influences on the data presented and we do not advocate for one device or company over another. All the data are freely available and the Monte-Carlo code is also available under request.

As lead author and address for correspondence KKW would point out that in both his clinical and academic work in heart failure and devices he is exposed to and has worked with all pacemaker manufacturers. Moreover, whilst JRB is employed by a device company, the manuscript makes no comment positive or negative on that company’s devices. The overriding message of our manuscript is that longevity can be assessed reliably using the new approach.

Minor Points

1. The PCI formula and its derivation should be described earlier in the text and more intuitively (e.g., through a flowchart).

Response: The rational for PCI is now better explained in supplementary 1.

2. Several instances of placeholders (“Error! Reference source not found.”) remain and must be corrected.

Response: We apologise for this failure of the PDF conversion to include bookmarks and cross references and have corrected this.

3. Some essential figures referenced (e.g., Figures 1–3) lack clarity or are difficult to interpret without captions. Ensure all figures are embedded with standalone readability.

Response: Thank you for this. The captions have been added.

4. Maintain consistency between device types (e.g., VVI vs. leadless SR), and clarify acronyms at first mention.

Response : We have checked and adjusted anywhere where ‘VVI’ was used to describe hardware. This occurred exclusively during our description of leadless devices where the convention is somewhat less embedded since DR is in some ways a misnomer given that to provide DDD pacing, one needs two devices. Nevertheless, we have taken the conventional approach and applied it to capsules.

5. Clarify rationale behind simulation parameters, especially pacing thresholds and outputs. Are they derived from actual patient data?

Response : The method section and now also Supplementary Material 2 describe the sources of the data and that they are all based on literature review. References are included in both places.

Suggested Additional Experiments
To enhance the robustness and applicability of the manuscript, the following experimental extensions are recommended:

1. Apply PCI in a prospective cohort where device programming and longevity outcomes are tracked, validating its predictive capacity beyond historical data.
2. Conduct benchtop measurements for key programming features (e.g., automatic capture management, aCRT) to empirically validate modeled current assumptions.
3. Analyze how real-time remote monitoring logs correlate with PCI-predicted consumption, offering a feedback loop for future model refinement.
4. Include example cases where device selection changed based on PCI, contrasting them with conventional choice outcomes.

Response: We are grateful to the Expert Reviewer and agree entirely. All these suggestions are completely relevant. They have been integrated into the article as next steps.

**References**

1. Udo EO, van Hemel NM, Zuithoff NP, Barrett MJ, Ruiter JH, Doevendans PA, Moons KG. Incidence and predictors of pacemaker reprogramming: potential consequences for remote follow-up. *Europace* 2013;**15:**978-83. doi: 10.1093/europace/eut002
2. Thibault B, Ducharme A, Baranchuk A, Dubuc M, Dyrda K, Guerra PG, Macle L, Mondésert B, Rivard L, Roy D, Talajic M, Andrade J, Nitzsché R, Khairy P; CAN‐SAVE R Study Investigators. Very Low Ventricular Pacing Rates Can Be Achieved Safely in a Heterogeneous Pacemaker Population and Provide Clinical Benefits: The CANadian Multi-Centre Randomised Study-Spontaneous AtrioVEntricular Conduction pReservation (CAN-SAVE R) Trial. *J Am Heart Assoc* 2015;**4:**e001983. doi: 10.1161/JAHA.115.001983
3. Stockburger M, Defaye P, Boveda S, Stancak B, Lazarus A, Sipötz J, Nardi S, Rolando M, Moreno J. Safety and efficiency of ventricular pacing prevention with an AAI-DDD changeover mode in patients with sinus node disease or atrioventricular block: impact on battery longevity-a sub-study of the ANSWER trial. *Europace* 2016;**18:**739-46. doi: 10.1093/europace/euv358
4. Auricchio A, Ellenbogen KA. Reducing Ventricular Pacing Frequency in Patients With Atrioventricular Block: Is It Time to Change the Current Pacing Paradigm? *Circ Arrhythm Electrophysiol* 2016;**9:**e004404. doi: 10.1161/CIRCEP.116.004404
5. Gadler F, Valzania C, Linde C. Current use of implantable electrical devices in Sweden: data from the Swedish pacemaker and implantable cardioverter-defibrillator registry. *Europace* 2015;**17:**69-77. doi: 10.1093/europace/euu233
